# Sun Protection Beliefs among Hispanics in the US

**DOI:** 10.1155/2014/161960

**Published:** 2014-11-09

**Authors:** Marimer Santiago-Rivas, Chang Wang, Lina Jandorf

**Affiliations:** Department of Oncological Sciences, Cancer Prevention and Control Division, Icahn School of Medicine at Mount Sinai, One Gustave L. Levy Place, New York, NY 10029-6574, USA

## Abstract

*Purpose*. We reviewed the literature on sun protection beliefs in Hispanics living in the United States to explore what challenges are faced by area of research. *Method*. A review of PubMED, PsycINFO, and CINAHL databases was performed. Studies were published in peer-reviewed journals (in all years available) and written in English. The search terms used were [“skin cancer” OR “sun protection”] AND [“Latino” OR “Hispanic”] AND “beliefs.” Eligible papers were included in the final analysis after meeting the following inclusion criteria: (1) the records had to quantitatively examine and report sun protection beliefs in Hispanics, (2) the number of Hispanic participants in the sample had to be clearly specified, and (3) studies reporting differences in sun protection beliefs between Hispanics and other racial and ethnic groups were included in the review. *Results*. Of the 92 articles identified, 11 met inclusion criteria and addressed sun protection beliefs regarding skin cancer seriousness and susceptibility, and benefits and barriers of sun protection and skin cancer risk behaviors. Characteristics of studies and results were examined. *Conclusion*. There is insufficient evidence to determine a pattern of sun protection beliefs among Hispanics in the United States. More quality studies are needed which focus on sun protection beliefs in Hispanics.

## 1. Introduction

Skin cancer is the most common cancer in the United States (US). It is estimated that close to 4 million skin cancer diagnoses (including basal cell and squamous cell carcinomas) are made every year [1]. Melanoma (an aggressive form of skin cancer) is diagnosed in more than 70,000 persons every year, creating a high health and economic burden with an estimated annual cost of $3.5 billion [2]. Risk factors for skin cancer include sun sensitivity (sunburning easily, difficulty tanning), a history of excessive sun exposure, sunburns, use of artificial tanning, and a past history of skin cancer [1]. Most of skin cancer cases could be prevented by protecting the skin from excessive sun exposure and avoiding indoor tanning. Results from an analysis of national data showed that the majority of the US population reported infrequent incidence of sun protection behaviors [3]. Characteristics of groups reporting lower incidence of sun protection include being young (under the age of 40), having a lower education level, being a smoker or a risky drinker, and being less sensitive to the sun [3]. Health research should focus on the identification of psychosocial and modifiable variables to promote sun protection among groups at higher risk for skin cancer and in the general population.

Even when it has been documented that the Hispanic/Latino (referred to as Hispanic) population suffers from a disparity regarding certain cancers compared to non-Hispanic whites (referred to as whites), the lifetime risk of developing skin cancer is higher among whites than other racial groups. For melanoma, it is higher among whites (2.9% in men, 1.9% in women) than in Hispanics (0.52% in men, 0.51% in women) [1, 4]. A study conducted in Miami showed that, among 3000 cases of nonmelanoma skin cancer reviewed, 60.1% were diagnosed in whites and 38.4% were diagnosed in Hispanics [5]. Findings using the Southeastern Arizona Skin Cancer Registry showed that the rates for nonmelanoma skin cancer in whites were approximately 11 times greater than rates for Latinos [6]. A case control study of nonmelanoma cancer diagnoses in Hispanics (with whites as control) showed that 15.3% of Latino patients reported recurrence of their malignancy as compared to 31.3% of controls [7]. Also, a lower proportion of Latinos (34.0% versus 61.3% controls) had a current diagnosis or prior history of actinic keratosis. On the other hand, skin cancer has been associated with considerable morbidity and mortality in the Hispanic population. Compared with whites, Hispanics have lower 5-year melanoma survival rates, 76.6% versus 87.0% for men and 88.3% versus 92.3 for women [4]. Hispanics are more likely to have advanced and thicker melanomas at diagnosis when compared with whites [8–16]. A greater percentage of melanomas occurred among Hispanics in younger age groups (24.4% less than 40 years old) compared with blacks and whites, 15.8% and 14.3%, respectively [16]. Also, Hispanics tend to report lower frequency of skin-related visits to dermatologists than their white counterparts [17]. Data obtained from cancer registries of Puerto Rico, New York, New Jersey, and Connecticut show that Puerto Ricans living in the US report higher melanoma rates than those residing in Puerto Rico [18]. At the same time, there are variations in the behaviors reported by Hispanics and non-Hispanics. A systematic review examined the incidence of sun protection behaviors among Hispanics in the US [19]. Overall, the prevalence of these behaviors is both low and mixed. While a slightly lower share of Hispanics (9.5–29.9%) report usage of sunscreen either most of the time or always compared to 16.5%–35.9% of whites, Hispanics reported slightly higher rates of wearing hats either most of the time or always (23.9–25.0% versus 20–20.7%). Recent studies of sun protection behaviors show that around 53% of Hispanics stay in shade, and around 20% use protective clothing when outside on a warm sunny day either most of the time or always [20, 21]. Hispanics who are less acculturated report lower rates of sunscreen use than those who are more acculturated [21]. Still, little is known about skin cancer risk factors in the Latino population. It is critical to identify psychosocial and modifiable factors influencing skin cancer morbidity and mortality in Hispanics in the US.

The Community Preventive Service Task Force reviewed skin cancer prevention evidence from a Community Guide systematic review published in 2004 combined with more recent evidence [22, 23]. The review found that education interventions in primary and middle schools (Kindergarten–8th grade), which include strategies to integrate parents, caregivers, and teachers, decrease sun exposure, sun protection, and formation of new moles. Multicomponent, communitywide interventions including a combination of individual-directed strategies (e.g., activities to change the knowledge, attitudes, beliefs, or behaviors), mass media campaigns, and policy changes are recommended based on evidence of effectiveness in increasing sunscreen use, but results for effects on other protective behaviors are mixed. Results also suggest benefits in reducing sunburns among children. In addition, findings illustrate that other approaches, such as mass media alone, provider education and media-based education sessions in health care settings, and educational activities in high school and colleges, did not provide sufficient evidence to determine their applicability for skin cancer prevention. Many of these studies were conducted outside of the US (i.e., Australia and the United Kingdom), but the Task Force suggests that findings are likely to be applicable to the US because results were similar across countries. Various interventions and education initiatives in the recent past have targeted minorities with the intention of improving skin cancer, but these were not multicomponent initiatives [24–26]. A group of Hispanic women evaluated two educational videos to increase positive sun protection beliefs and behaviors [24]. There was an effect in skin cancer risk awareness postintervention, and participants reported they preferred the video emphasizing the benefits of sun protection for skin cancer prevention more than the video emphasizing its effect on photoaging. Little research has examined the association between sun protection behavioral outcomes and the health outcome of interest, that is, skin cancer incidence [23]. More research is needed to verify the efficacy of multicomponent, communitywide interventions addressing the effect of sun protection attitudes, perceptions, beliefs, and behaviors on increasing sun protection. In addition, research should evaluate its effect on decreasing sunburns (short-term effect) and skin cancer incidence (long-term effect) in the general public and in subgroups at particular risk for skin cancer.

This paper examines published studies that include health beliefs concerning skin cancer prevention and sun protection in Hispanics.

## 2. Materials and Methods

### 2.1. Search Strategy

We performed a search of the databases PubMed, PsycINFO, and CINAHL. All publication years and all search fields were included. The search was limited to articles in English and employed specific search keywords. One example of a search strategy used with the PubMED database is ((skin cancer) AND Hispanic) AND beliefs; ((skin cancer) AND Latino) AND beliefs; ((sun protection) AND Hispanic) AND beliefs; ((sun protection) AND Latino) AND beliefs. We decided to use broad search terms to make sure we would identify as many pertinent studies as possible. For our search, we decided to use the word “Hispanic” and “Latino” to indicate our population of interest, that is, US residents of Mexican, Cuban, Puerto Rican, Central American, South American, and other Spanish-speaking country origins. A search in PubMED demonstrated how research, with some exceptions (including US Census data and self-report), interchangeably uses these terms and lacks stratification of the members of this group [27]. A study by the Pew Hispanic Center found that more than half Hispanics (51%) have no preference for any of the two terms to describe their ethnicity [28]. At the same time, the term “Hispanic” was chosen to be used in this paper given that sun protection research applies this term more frequently compared with the term “Latino” (see Table 1 for list of the term(s) for ethnicity and raced used by each study included in this review). A manual secondary search of all bibliographies from relevant articles was performed to yield further relevant publications. We excluded studies conducted outside the US, as well as studies without data for Hispanic participants on the report of sun protection beliefs. Studies that compared the differences in sun protection beliefs between Hispanics and non-Hispanics were included as well.

### 2.2. Eligibility Criteria

Articles were reviewed for relevance with the criteria for inclusion being as follows. (1) The reports had to quantitatively examine and report (frequency, means, percentages, effect sizes, and/or odds ratio) sun protection beliefs in Hispanic samples, including constructs such as skin cancer risk/susceptibility and severity/seriousness, and sun protection beliefs (barriers and benefits of sun protection and skin cancer risk). (2) The number of Hispanic participants in the sample had to be stated. (3) Studies that reported the differences in sun protection beliefs between Hispanics and other racial and ethnic groups were used. Books, book chapters, meta-analyses, comments, and reviews were excluded.

## 3. Results

**Figure 1:**
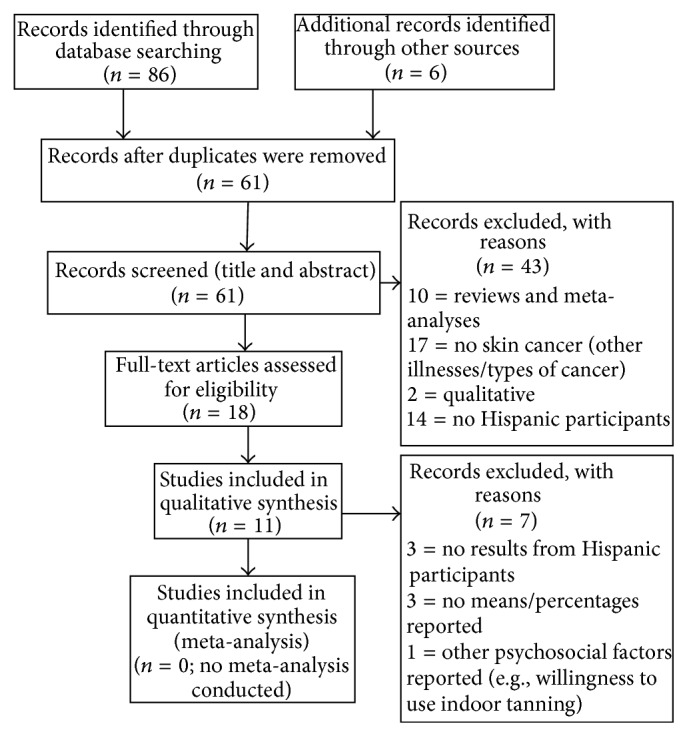
Flow diagram of literature search.

The first database searched was PubMED, followed by a search of PsycINFO and CINAHL. A total of 86 articles were identified from these databases, and 6 articles were identified from bibliographies, for a total of 92 records (see Figure 1). A search of duplicates was conducted, leaving 61 records that were title- and abstract-screened and 18 records that were screened in full. The title and abstract screening step evaluated the title and the abstract of each of the 61 articles to determine whether the abstracts met the following criteria: (1) informed about sun protection beliefs, (2) informed about the sample used (humans), (3) suggested that the study included Hispanics in the sample, (4) used English as publication language, and (5) indicated that the publication was peer-reviewed. As part of the full screening step, the manuscripts from the 18 abstracts were obtained and read in full to determine whether they met the eligibility criteria: (1) the records had to quantitatively examine and report sun protection beliefs in Hispanics, (2) the number of Hispanic participants in the sample had to be clearly specified, and (3) studies reporting differences in sun protection beliefs between Hispanics and other racial and ethnic groups were included in the review. These manuscripts were read in full, and eleven were included in the final analysis (see Figure 1). Data on the author, year of publication, sample characteristics, methodology used, measures selected, and quantitative results from each of the articles were abstracted and evaluated.

Findings are illustrated in Table 1. Six studies included adult participants, and three studies included children and adolescents (students in middle school and high school). One study included both adolescents and adults, and one study did not report information regarding the inclusion of participants that were under 18 years old (participants were patients at a dermatology clinic). Three studies excluded participants with history of skin cancer, and five studies reported data on sun protection behaviors. One study was population-based. Two studies had survey materials available in Spanish, and one study was conducted entirely in Spanish. Two studies reported data on country origin/heritage. Five studies had relatively small Hispanic samples (less than 100 participants). Most studies (*n* = 10) were published during the last decade (2004–2014).

Skin cancer seriousness (severity, worry) was a belief considered in three studies [29–31]. Results from a population study showed that Hispanics and whites share similar levels of worry about skin cancer (*P* > 0.05) [29]. One study reported a midrange score in terms of skin cancer worry in Hispanics. Another study found that perceived skin cancer severity was associated with incidence of total body examination (i.e., a head-to-toe examination of the skin performed by a physician used to identify suspicious growths that may be cancer or growths that may develop into skin), but not with incidence of skin self-examination (i.e., a head-to-toe examination of the skin performed by the individual, not a physician) [30, 31].

Most studies included in the review (*n* = 8) considered skin cancer susceptibility (or risk) beliefs. One study found that Hispanics believe they have lower than average risk of developing skin cancer, and that their level of risk is lower when compared with whites [32]. A second study reported that most Hispanics described their skin cancer susceptibility as average [33]. A third study also found lower perception of skin cancer risk when Hispanics were compared with whites but the difference was not significant when participants were asked to compare their own likelihood of getting skin cancer compared with the risk of an average person of the same age [29]. Two studies reported similar scores on their skin cancer risk and photoaging (changes in skin appearance induced by sun exposure) concern measures but used different scales for the scores [30, 34]. Participants were inclined to not agree or disagree (midrange score) with the following statement: “The natural color of my skin protects me from the sun” [30]. After quantifying qualitative information from an all-female sample, it was found that less than half of the participants believe they can develop skin cancer [24]. On the other hand, almost all participants were concerned about the effect of sun exposure on their appearance. Using a different sample as part of an experiment within the same study, participants reported an increase in skin cancer susceptibility “of Hispanics with fair skin and Hispanics with dark skin” (not their own susceptibility) after watching an educational video about sun protection behaviors. Another study asked Hispanics about the skin cancer susceptibility of people “in darker skin types,” and results showed that a slightly lower proportion of Hispanics (78%) endorsed this statement compared with white (91%) and black (86%) participants [35]. Another study reported that perceived skin cancer risk is associated with skin self-examination [31].

A strong effect size was reported for association between perceived peer norms for sun exposure and barriers to sun safety in Hispanic middle school students [36]. Hispanics are more likely to believe that there is not much they can do to lower their risk of getting skin cancer and that there are too many recommendations to prevent this illness [26]. It was also reported that more than half of Hispanics believe that tanning makes people look more attractive and do not endorse the belief that tanning makes people older [37]. One study showed that Hispanics tend to marginally agree more with statements regarding sun protection benefits than barriers [30]. Participants also indicated what were the most important benefits and barriers to engage in sun protection behaviors, with “avoid getting sunburn” and “not part of my daily routine” as frequently endorsed statements. Another study showed a similar pattern in terms of sun protection beliefs [34]. An additional reason Hispanics endorse for failing to use sunscreen is because they consider themselves “dark skinned” [38].

## 4. Discussion

This study examined published reports of sun protection beliefs in Hispanics, and we found eleven manuscripts that followed the established criteria. Results suggest that low skin cancer susceptibility is commonly found in this population and that Hispanics moderately perceive skin cancer as a serious health threat. Results also suggest that assessments of sun protection barriers and benefits vary significantly by study. Overall, findings illustrated that there are limited studies on psychosocial and modifiable factors that influence sun protection. Many of the studies included in this review have limited sample size or used samples that do not represent the heterogeneous Hispanic population (e.g., 70% of participants in one study were of Mexican origin) [30, 31]. Findings must be validated in larger, more comprehensive studies. Results also emphasize the need for comparable and consistent assessment regarding sun protection. This finding is consistent with previous skin cancer prevention results. An evaluation of interventions designed to educate primary care physicians about skin cancer showed a lack of uniformity across interventions and outcome assessments, preventing the direct comparison of intervention efficacy and the dissemination of effective components [39].

Skin cancer can be prevented by practicing sun protection, but skin cancer disparities might be associated with the perceptions, knowledge, attitudes, and beliefs Hispanics hold regarding skin cancer and sun protection. It has been found that individuals who express the benefits of sun protection are likely to report sun protection behaviors consistently more than those who communicate the barriers of sun protection [40–42]. Hispanics are more likely to believe there is little they can do to lower their chances of getting skin cancer, that there are so many recommendations about skin cancer prevention that they do not know which one to believe and believe that they are below average risk for skin cancer compared with whites. It is critical to understand the sets of beliefs that underlie sun protection among Hispanics and improve health promotion initiatives to decrease sun protection disparities.

Most common types of skin cancer are squamous cell carcinoma and basal cell carcinoma (SCC and BCC, resp.; nonmelanoma), and melanoma. These cancers have different causes and presentations. Basal cell carcinoma diagnoses are more common in Hispanics than squamous cell carcinoma and melanoma diagnoses [43, 44]. While person characteristics (e.g., light skin, sun sensitivity, and blistering sunburns early in life) and intermittent sun exposure are strong risk factors for the diagnosis of melanoma, both cumulative and intermittent sun exposure are the most common cause of basal cell carcinoma. In terms of presentation, melanoma usually involves sites not exposed to the sun, including palmar, plantar, and mucosal surfaces, and the lower extremities [45]. Areas such as the head and the neck regions seem to be more prone for basal cell carcinoma. Literature in sun exposure at the workplace indicates an elevated risk for SCC but is less conclusive for BCC [46]. A population-based control-study among individuals diagnosed with invasive melanoma found that frequent sunscreen use when not planning to be in the sun during the last 20 years was strongly associated with lower likelihood of melanoma [47]. Also, those who reported use of sun protection (not sunscreen) were at lower risk of developing melanoma, even if its use was inconsistent. Consistent with the compensation hypothesis of sunscreen use and increased sun exposure, optimal use of sunscreen SPF+15 was associated with highest amount of sun exposure. Research directly associating sun protection behaviors to decreased skin cancer risk is limited and inconsistent. The present study shows that research still struggles to investigate and understand the specific factors that might be associated with melanoma and nonmelanoma skin cancer incidence, and disparities in skin cancer. Research should clarify the association between the disease, the target population, and the particular mechanisms to prevent the disease.

Previous research shows a moderate level of awareness about skin cancer risk factors and prevention behaviors among Hispanics. Using a qualitative approach, forty Hispanics were asked about their understanding of skin cancer risk terminology [26]. Results illustrated that participants did not recognize possible indicators of skin cancer risk (e.g., painful sunburns). One study showed that more Hispanics do not use sunscreen because they perceive themselves as dark skinned when compared with whites and Asian/Pacific Islanders (29% versus 3% versus 11.4%, *P* < 0.05) [38]. Results included in the present review emphasize the need for improved assessments of sun protection beliefs and to incorporate the evaluation of the meaning and significance Hispanics give to sun protection: do they know what protective clothing is?; what do they think about using sunscreen “all the time” when out in the sun, including cloudy days?; would they wear a hat at all?; do they know how their skin reacts to sun exposure? These are questions that future research should address if we want to report a more accurate analysis of sun protection. This review underscores the importance of developing culturally relevant, validated, and reliable measures of sun protection and skin cancer risk perception for Hispanic adults, adolescents, and children.

Our search was limited to peer-reviewed journals, which generally publish studies with significant results. It was also limited to results on sun protection beliefs in Hispanics living in the US. Findings cannot be generalized to studies conducted in other countries, and there is a possibility that important elements of sun protection were not captured in our review. A strength of this review is that it helped us realize that the full potentials of the assessment and applications of sun protection beliefs for the prevention of skin cancer in Hispanics remain largely unverified and untested. This finding should open the doors to many research initiatives to promote health in a growing minority population and to identify and understand factors that contribute to disparities in the incidence and mortality of cancer.

Hispanics are a diverse group that exhibit differences in terms of sun protection behaviors, sun sensitivity, level of acculturation, country of origin, access to health services, and socioeconomic status. Future research should develop comprehensive, culturally sensitive measures of sun protection beliefs, facilitators, and barriers. Measures should be grounded in theory, research evidence, and ethnographic study. Also, researchers must ensure that their recruitment strategy attains a more diverse sample than previous research. The National Cancer Institute states that overcoming cancer health disparities is one of the best opportunities we have for lessening the burden of cancer [48]. It is goal of the institute to improve the understanding of the causes of cancer health disparities as a way to eliminate them. It is critical to identify modifiable factors that can reduce skin cancer morbidity and mortality disparities in the US. There is a need for informed, culturally sensitive measures to assess sun protection in the Hispanic population in order to (1) directly inform the development of a study to investigate the ability of sun protection beliefs to predict the likelihood to engage in positive health outcomes (i.e., sun protection behaviors), (2) make informed assessments of the effect of sun protection on skin cancer morbidity and mortality, (3) contribute to the limited literature on sun protection in Hispanics, (4) inform the development of targeted public health recommendations and initiatives to increase sun protection, and (5) make a contribution to the identification and understanding of experiences Hispanics have regarding sun protection.

## Figures and Tables

**Table 1:** Findings related to sun protection and skin cancer risk beliefs in Hispanics.

Study	Sample	Design	Term used for ethnicity and race	Sun protection/skin cancer risk beliefs and findings (Hispanic)	Comments about quality of study
Andreeva et al. (2008) [36]	Total (*N* = 1,782) Hispanic (*N* = 437) *Adolescents *	Cross-sectional self-survey (no information about survey in Spanish)	Hispanic non-Hispanic	Results affect size in structural model (standardized solution): perceived peer norms for sun exposure and barriers to sun safety (0.459); barriers to sun safety and protanning attitudes (0.015), and barriers to sun safety and sun-safe behavior (0.142). Hispanics having lower scores on the tan-related measures and slightly higher scores on barriers.	Data were collected as part of sunny days, healthy ways program in Colorado, New Mexico, and Arizona. Information about validation of measurements in Hispanic sample was not reported. Original study reported low alpha reliability (less than 0.70) for measures of barriers to sun protection and peer norms for sun exposure.

Buster et al. (2012) [29]	Total (*N* = 1,246) Hispanic (*N* = 161) *Adults *	Population-based interviewer survey (no information about interview in Spanish)	Hispanic black, white	Results comparison with whites: likelihood of future skin cancer (OR 1.41); likelihood of skin cancer compared with average person of the same age (OR 0.83); worry about skin cancer (OR 0.83); there is not much you can do to lower chance of getting skin cancer (OR 3.87); there are so many recommendations about preventing skin cancer; it is hard to know which ones to follow (OR 3.35).	Data collected by Health Information National Trends Survey (HINTS). Statistical analyses were conducted with samples of different sizes (Hispanic *n* = 161 versus white *n* = 966). The HINTS is available in both Spanish and English (not mentioned in the paper), but there was no mention of possible influence of language preference.

Cheng et al. (2010) [37]	Total (*N* = 1,214) Hispanic (*N* = 266) *Adolescents *	Cross-sectional self-survey (no information about survey in Spanish)	Hispanic black, white	Percentages: tanning makes people look more attractive (true 61%); tanning makes people look older (True 27%).	The survey was part of an educational intervention which included a pretest, a 30-minute lesson on sun protection, and a posttest. There was no mention of this intervention being available in Spanish and English, or assessment of cultural competence. Limitations include a population surveyed from only New Jersey public schools and small Hispanic sample.

Coups et al. (2014) [30]	Total (*N* = 787 Hispanic) *Adults *	Cross-sectional self-survey/online (survey available in Spanish)	Hispanic	Weighted means (range 1–5) and standard deviations: suntan benefits = 2.58 (1.08); sunscreen benefits = 3.71 (0.94); shade seeking benefits = 3.62 (0.96); sun protective clothing benefits = 3.72 (0.94); sunscreen barriers = 2.51 (0.78); shade seeking barriers = 2.77 (0.71); sun protective clothing barriers = 2.70 (0.83); skin cancer worry = 2.51 (1.13); perceived skin cancer risk = 3.71 (1.06); photoaging concerns = 3.72 (1.02); perceived natural skin protection = 2.61 (1.26).	Participants completed an English- or Spanish-language online survey. Information about validity and reliability of measures by language preference was not provided. Authors mentioned survey items that were not already available in Spanish were translated, affecting the cultural appropriateness of the study for the Spanish-speaking Hispanic population. A large proportion of the sample was of Mexican origin (71%).

Coups et al. (2013) [21, 31]	Total (*N* = 787 Hispanic) *Adults *	Cross-sectional self-survey/online (survey available in Spanish)	Hispanic	Correlates of sun protection beliefs and skin self-examination (and total body examination): perceived skin cancer risk AOR 1.34 (AOR 0.89); perceived skin cancer severity AOR 1.05 (1.91).	Participants completed an English- or Spanish-language online survey. Information about validity and reliability of measures by language preference was not provided. Participants' state of residence was as follows: California, *n* = 379; Texas, *n* = 231; Florida, *n* = 110; Arizona, *n* = 41; and New Mexico, *n* = 26.

Heckman and Cohen-Filipic (2012) [34]	Total (*N* = 74 Hispanic) *Adolescents and young adults *	Cross-sectional self-survey (survey available in Spanish)	Hispanic	Means (range 0–10 and 4–20) and standard deviations: how likely is it that you will develop skin cancer? = 3.70 (2.43); how likely is it that your skin will age too soon? = 3.69 (2.62); benefits of UV exposure (four items) = 11.10 (3.83); benefits of sun protection (four items) = 13.11 (3.87).	This pilot study was part of an educational collaboration between a high school science department and a cancer center in the suburbs of Philadelphia. A small Hispanic sample (*n* = 74) was studied. Items from original study were developed from a sample of parents and children (*N* = white = 55%, African American = 26%, and Hispanic = 15%). The sun protection benefits scales were originally developed as mixed sun protection knowledge and attitude scale, and it had low alpha reliability (less than 0.70). The reviewed study showed better reliability. Participants could choose to receive information and surveys in either English or Spanish, but no information about appropriateness of the measures for culture or language preference was provided.

Hernandez et al. (2014) [24]	Total study 1 (*N* = 52 Hispanic); total study 2 (*N* = 80 Hispanic; 67 women, 13 men) *Adults *	Qualitative (quantitative results reported); experimental phase (video) (study conducted in Spanish)	Hispanic	Study 1 frequencies: believes she can develop a skin cancer (Yes = 25/52); concerned about lentigines (Yes = 48/52) and wrinkles (Yes = 35/52). Study 2 frequencies: fair-skinned Hispanics are at risk for skin cancer (prevideo, agreement = 54/80; postvideo agreement = 72/80); dark-skinned Hispanics are at risk for skin cancer (preagreement = 44/80; postagreement = 69/80).	Effect of two short Spanish-language films on sun protection beliefs was tested (in Chicago, Illinois). One emphasized photoaging benefits of sun protection, while the second focused on its benefits for skin cancer prevention. Nine patients at a dermatology clinic, whose primary language was Spanish, were asked to view the videos and review the questionnaires before it being administered. The samples studied were small, and it is not clear how results would apply to English-speaking Hispanics. Authors developed the videos using primarily the opinions of women rather than men, and the male sample size in the intervention group was limited.

Imahiyerobo-Ip et al. (2011) [35]	Total (*N* = 165) Hispanic (*N* = 38) *Patients *	Cross-sectional self-survey (no information about survey in Spanish)	Hispanic white, African American, Asian, and others	Frequency and percentage: believes that skin cancer can happen in darker skin types (29/37; 78%).	A survey was administered to 165 patients seeking care from a dermatology practice in New York City. Limitations include the small sample size and the inclusion of patients who may have had a history of actinic keratoses.

Ma et al. (2007) [32]	Total (*N* = 369) Hispanic (*N* = 221) *Adolescents *	Cross-sectional self-survey (no information about survey in Spanish)	White Hispanic white non-Hispanic	Frequencies, percentages, and results comparison with whites: chances of developing skin cancer in the future “higher than average” (4.1%), “average” (19%), “lower than average” (51.6%), and “don't know” (25.3%). Logistic regression “average or above” (OR 0.6) after controlling for age, sex, skin type, and family history of skin cancer.	A pilot survey study using 1 of the 33 public high schools located in the Miami-Dade County area of Florida. A self-administered, anonymous survey, which was derived from a tool used in a derivate of the national Nurses' Health Study (94% white). Information about validation of measurements in Hispanic sample, or clarification of the term “average risk of skin cancer” was not reported.

Mahler (2014) [38]	Total (*N* = 1183) Hispanic (*N* = 65) *Adults *	Baseline self-survey (no information about survey in Spanish)	Hispanic white, Asian/Pacific Islander	Significant differences in percentages of responses when compared with whites after controlling for skin sensitivity. Sunscreen benefits: avoid getting too dark (Hispanic 15.1%, white 5.8%). Sunscreen barriers: it is too much trouble (Hispanic 16.4%, white 30.6%); I am dark skinned (Hispanic 29.1%, white 3%).	The data were drawn from baseline questionnaires completed during 9 different sun protection experiments conducted in San Diego, California. Participants who indicated ever using sunscreen checked any of the listed sunscreen benefits/barriers. The authors mentioned that the list was developed through piloting, but no additional information is provided. No information about appropriateness of the measures for culture or language preference was provided. A small Hispanic sample was used for the statistical comparisons.

Pipitone et al. (2002) [33]	Total (*N* = 153) Hispanic (*N* = 27) *Adults *	Cross-sectional self-survey (no information about survey in Spanish)	White Hispanic white non-Hispanic	Frequencies perceived risk of melanoma or skin cancer “higher than average” (4%), “average” (59%), “lower than average” (22%), and “don't know” (15%).	Prospective survey of a group of suburban employees to evaluate perceptions of skin cancer risk. Low participation in the study and small Hispanic sample. Information about validation of measurements in Hispanic sample, or clarification of the term “average risk of skin cancer” for comparisons was not reported.

OR: odds ratio; AOR: adjusted odds ratio.
